# Microbial diversity in drug-naïve Parkinson’s disease patients

**DOI:** 10.1371/journal.pone.0328761

**Published:** 2025-08-18

**Authors:** Eliša Papić, Valentino Rački, Mario Hero, Ana Nyasha Zimani, Mojca Čižek Sajko, Gloria Rožmarić, Nada Starčević Čizmarević, Saša Ostojić, Miljenko Kapović, Goran Hauser, Aleš Maver, Borut Peterlin, Anja Kovanda, Vladimira Vuletić

**Affiliations:** 1 Department of Neurology, Faculty of Medicine, University of Rijeka, Rijeka, Croatia; 2 Clinic of Neurology, Clinical Hospital Center Rijeka, Rijeka, Croatia; 3 Clinical Institute of Genomic Medicine, University Medical Centre Ljubljana, Slovenia; 4 Department of Medical Biology and Genetics, Faculty of Medicine, University of Rijeka, Rijeka, Croatia; 5 Department of Internal Medicine, Faculty of Medicine, University of Rijeka, Rijeka, Croatia; 6 Clinical Hospital Center Rijeka, Rijeka, Croatia; 7 Faculty of Medicine, University of Ljubljana, Ljubljana, Slovenia; Mae Fah Luang University School of Anti in Aging and Regenerative Medicine, THAILAND

## Abstract

Parkinson’s disease (PD) is a neurological disorder characterized by rigidity, bradykinesia and tremor. Several genetic and environmental causes of PD are known, and there is emerging evidence of the possible contribution of the gut microbiome to the disease onset, severity, and response to therapy. While previous research has shown several differences in the microbiome of PD patients under therapy as opposed to healthy controls, few prospective studies have included drug-naïve patients. In order to evaluate the gut microbiome composition prior to therapy initiation, we collected and performed 16S rRNA gene sequencing of the stool samples from 49 drug-naïve PD patients and compared them to 34 diet and lifestyle-matched controls from the Croatian population (GiOPARK Project). While no significant alpha diversity difference was observed between the patients and controls, the differential relative abundance analysis showed an increase in *Bacteroides fluxus*, *B. interstinalis*, *B. eggerthii*, and *Dielma fastidiosa* in the drug-naïve PD patients compared to controls, while *Alistipes*, *Barnesiella* and *Dialister spp*. were decreased in patients compared to controls. Despite preserved overall diversity, these changes may indicate early microbial dysbiosis and represent a foundation for future studies exploring microbiome changes across disease progression and treatment.

## Introduction

Parkinson’s disease (PD) is a neurodegenerative disease presenting with the classical triad of symptoms, which include rigidity, bradykinesia, and tremor [1], while also including a wide range of other motor and non-motor symptoms [2]. The primary pathophysiological mechanism is thought to lie in the accumulation of α-synuclein in the central nervous system, first in the substantia nigra, and then throughout the brain. This leads to the progressive loss of dopaminergic neurons, which in turn slowly leads to severe motor and cognitive impairment [3]. It is a multifactorial condition with genetic and environmental causes, with microbiota recently identified as a possible environmental contributor [4].

Microbial composition has been shown to differ in PD patients compared to the healthy population. The observed differences have been proposed to be involved in the pathological retro-axonal transport potentially responsible for α–synuclein propagation into the central nervous system, even leading to prodromal symptoms such as hyposmia and gastrointestinal dysfunction [5]. Changes in the microbiome have also been implicated in motor symptom severity in later stages of the disease [6] and response to PD therapy, most notably levodopa [7].

These changes could potentially play an important future role in the specific therapy of PD, since targeting the microbiome through various therapeutic approaches could modulate disease course and severity. These approaches include prebiotics [8] and probiotics [9], dietary changes [10], and fecal matter transplantation (FMT) among others [11]. Antibiotics are also known to alter the gut microbiome [12], and certain antibiotics, such as rifampicin, have shown a beneficial effect in PD [13], despite potentially inducing dysbiosis, even long after cessation [14].

It is thought that the microbiota’s role is most impactful in the prodromal, early stages of the disease [15], with the gut-brain axis acting as an essential mechanism through which microbiota could influence disease onset and progression [16]. Various potential pathways have been postulated by which this complex bidirectional system could operate, including the trans-neuronal propagation of α–synuclein through the vagal nerve [17,18], as well as loss of protective metabolic products such as short-chain fatty acids (SCFA), which are secreted by microbiota [15]. It has been hypothesized that there are etiologically different subtypes of PD based on the initial location of Lewy body pathology, with the ‘body-first’ placing it in the enteric nervous system and the ‘brain-first’ placing it in the brain. Research already showed a distinct microbiota profile in “body-first” PD patients as opposed to “brain-first” PD patients and healthy controls, with an increase in abundances of taxa such as *Escherichia coli* and *Akkermansia municiphila* and a decrease of taxa producing SCFA [19].

Despite these recent advances in our understanding of the interplay of the microbiome and PD, as we recently reviewed [20], only a limited number of studies have so far prospectively examined the differences in microbiome composition and metabolic function and their effect on motor and non-motor severity in PD and the microbiome composition changes over time under specific PD therapy. Therefore, determining the gut microbiome of drug-naïve PD patients before therapy implementation represents a valuable baseline dataset for further studies.

With this aim in mind, as part of our prospective epidemiological study of PD in Croatia (GiOPARK Project), we have enrol led 54 drug-naïve PD patients and 34 healthy controls to compare their gut microbiome composition using 16s rRNA sequencing.

## Materials and methods

### Ethics statement

The study was approved by the Ethical Committee of the Medical Faculty in Rijeka, Croatia (2170-24-04-3-19-2) as well as the Ethical Committee of the Clinical Hospital Centre in Rijeka, Croatia (2170-29-02/1-19-2) and was conducted according to the principles in the Declaration of Helsinki. All individuals included in the research provided written informed consent before recruitment.

### Sample collection and clinical assessment

Patient and control selection, as well as sample collection was conducted at the Clinic for Neurology, Clinical Hospital Centre in Rijeka, Croatia. Analysis was conducted at the Clinical Institute of Genomic Medicine, University Medical Centre Ljubljana, Slovenia.

The inclusion criteria for the study included a diagnosis of PD, made according to the Movement Disorder Society Clinical Diagnostic criteria, for which the patients have not yet started treatment (drug-naïve). The healthy control group was chosen from volunteers from the same geographical region to match the patients’ characteristics. Exclusion criteria for both groups included: adherence to special diets, chronic intestinal inflammation, any type of autoimmune disease, acute infectious diseases, acute active inflammation, medical history of major gastrointestinal surgery, active use of antibiotics, probiotics, nutritional supplements, corticosteroids, or other immunosuppressive drugs.

Initially, 54 PD patients were screened for the GIOPARK study. Two cases were excluded due to chronic inflammatory bowel conditions and three withdrew from participation. Finally, 16S rRNA Sequencing was performed for a total of 49 PD patients, and 34 healthy controls.

Upon initial examination, both groups filled out the following epidemiological and clinical questionnaires/ scales. The epidemiological questionnaire dealt mainly with daily and weekly intake of various produce, type of diet as well as other habits such as smoking, drinking and coffee consumption (S1 Table). In our analysis we focused mainly on whether or not our patients followed a Mediterranean diet plan. Besides those, we used the Holmes-Rahe Lifestyle Stress Inventory [21] as well as the NSAD (National Stress Assessment Day) Questionnaire [22]. We used the Unified Parkinson’s disease Rating Scale (UPDRS) [23] in both groups to test for motor and non-motor symptom severity. Non-motor symptoms were further evaluated through the Non-Motor Symptom Questionnaire [24] and cognitive status was evaluated through the Montreal Canada Cognitive Assessment (MoCA).

### Bacterial DNA isolation, 16S rRNA gene amplification and sequencing, and bioinformatics analysis

The gut swabs for genomic bacterial DNA isolation were transferred to the laboratory in the Amies transport medium and stored at −80°C until isolation.

Isolation was performed as previously described [25]. Briefly, mechanical and enzymatic lysis using lysozyme was performed as described by Ravel et al. [26]. Bacterial DNA was obtained from the lysate swab medium using QIAamp DNA Mini Kit (QIAGEN, Hilden, Germany) according to the manufacturer’s instructions for gut/stool sample extraction. DNA concentrations were measured using a Qubit dsDNA high-sensitivity assay kit (Thermo Fisher Scientific).

Bacterial 16S rRNA was amplified using PCR with an Illumina adapter containing primers 341F and 805R for the informative V3-V4 region, and the PCR products were visualized on an agarose gel before proceeding to clean-up and indexing.

The amplicons of each sample were labeled with Nextera XT Indexes, and sequencing libraries were prepared according to the standardized 16S Metagenomic Sequencing Library Preparation Protocol (Illumina®). Libraries of pooled samples were sequenced on the Illumina MiSeq sequencer according to the manufacturer’s specifications in the 2 × 300 bp pair-end runs (MiSeq Reagent kit v3).

The datasets generated for this study can be found in the Sequence Read Archive (NCBI) repository, BioProject PRJNA1196315, http://www.ncbi.nlm.nih.gov/bioproject/1196315.

### Bioinformatics analysis

Bioinformatics analyses were performed in the R environment version 4.3.3. using the Bioconductor workflow adapted from Callahan et al. [27].

Briefly, sequence data consisting of demultiplexed FASTQ files with at least 30 Phred scores were trimmed and filtered using DADA2 [28]. Trimming was performed on joint reads so that the first 17 bp of forward reads and the first 21 bp of reverse reads were trimmed to remove primers, and forward reads were truncated to 250 bp and reverse reads to 230 bp in length. No ambiguous base calls were allowed, and filtering parameters were set to a maximum of 2 expected errors per read pair. Quality scores and error rates were assessed separately for all runs to minimize batch effects resulting from run variability. All runs contained a balanced representation of PD and control samples.

Amplicon sequence variants (ASV) were inferred, and chimeric sequences were removed using DADA2 [28,29], as previously described [25,30]. Taxonomical classification of amplicon sequence variants was determined using the RDP version 18 [31] and Silva v138.1 [32]. Sequence, taxonomic and clinical data were combined into a single object using the phyloseq package for R (version 1.46.0) [33].

### Statistical analysis

Demographic and clinical characteristics were compared between patients and healthy controls. To assess the normality of the distribution of numerical variables, we used the Shapiro-Wilk test. Since none of the variables met the assumption of normality, we applied the Mann-Whitney U test to compare the distributions between the study groups. For evaluating the association between categorical variables and group membership, we employed either the chi-square test or Fisher’s exact test, as appropriate. Statistical analysis was performed using JASP software (University of Amsterdam, Netherlands) and version 4.1.3 of the R computing environment.

Statistical analyses of the microbiome were performed in R version 4.3.3 using the vegan package version 2.6−2 [34] and DESeq2 version 1.42.1 [35]. The prevalence threshold was set at 5% of the total samples. Rarefaction without replacement was performed to even depth [36]. The microbial richness and alpha diversity of the sample groups were visualized using Chao1 and the Simpson diversity index, as implemented in the phyloseq package (version 1.46.0) [33]. Beta diversity was visualized by generating Principal Coordinate Analysis (PCoA) using unweighted UniFrac as distance in the phyloseq and ggplot2 package for R [33,37].

The differential relative abundance of ASV and corresponding taxonomic groups was calculated using DESeq2 version 1.42.1 [35]. Samples were normalized using the Wald test, and the default Benjamini-Hochberg correction implemented in the DESeq function was used for multiple-inference correction.

## Results

### Characteristics of participants

We assessed the demographic and clinical parameters of our study population, which included 83 individuals (49 patients and 34 controls) using epidemiological and clinical questionnaires (Table 1).

**Table 1:** Demographic and clinical characteristics of PD patients and controls.

Demographic and clinical characteristics	PD (n = 49) n (%)	Controls (n = 34) n (%)	P-value
Sex, male	24 (49.0)	8 (23.5)	0.019^b^
Age	63.73 ± 11.94	46.47 ± 13.26	<0.001^a^
Positive family history	10 (20.4)	2 (5.9)	0.110^c^
Early onset of PD	5 (10.2)	/	/
Mediterranean diet	43 (87.7)	30 (88.2)	1^c^
Coffee consumption (at least 1x a day)	34 (69.4)	31 (91.2)	0.018^b^
Regular alcohol consumption	32 (65.3)	32 (94.1)	<0.001^b^
Wine	21 (42.8)	25 (73.5)	0.006^b^
Beer	13 (26.5)	15 (44.1)	0.096^b^
Smokers	4 (8.1)	13 (38.2)	0.001^b^
Average NSAD	8.10 ± 4.98	7.32 ± 4.80	0.207^a^
Average HRLSI	102.75 ± 101.33	80.00 ± 102.80	0.200^a^
**Clinical characteristics**			
Average UPDRS I	1.92 ± 1.68	0.67 ± 0.84	<0.001^a^
Average UPDRS II	7.79 ± 4.74	0.32 ± 1.45	<0.001^a^
Average UPDRS III	21.04 ± 11.37	0.97 ± 1.66	<0.001^a^
Average NMSQ	7.39 ± 5.21	3.29 ± 3.05	<0.001^a^
Average MoCA	25.94 ± 3.67	29.35 ± 1.02	<0.001^a^
Constipation	21 (42.8)	14 (41.1)	0.879^b^
Stool incontinence	5 (10.2)	0 (0)	0.075^c^
Incomplete defecation	9 (18.4)	3 (8.8)	0.343^c^

Numeric data are presented as means ± standard deviation and compared between the study groups using the Mann-Whitney test (marked as ^a^), while categorial data are presented as frequencies (%) and tested using either the chi-square test (marked as ^b^) or Fisher’s exact test (marked as ^c^). NSAD – NSAD Stress questionnaire, HRLSI – Holmes-Rahe Lifestyle Stress Inventory, UPDRS – Unified Parkinson’s disease Rating Scale, NMSQ – Non-Motor Symptom Questionnaire, MoCA – Montreal Canada Cognitive Assessment.

The patient group had a significantly higher number of men, a higher average age and expectedly, more PD-positive family history than controls. Regardless of group almost all participants adhered to the Mediterranean diet. Healthy controls reported significantly more regular coffee consumption, smoking and regular alcohol consumption (Table 1).

Gastrointestinal issues were more prevalent in the PD group, but did not reach statistical significance, likely reflecting the initial stage of disease (Table 1).

### 16S rRNA sequencing results

V3-V4 region 16S rRNA sequencing (MiSeq, Illumina) was performed on a total of 83 samples (49 PD and 34 controls), as previously described [38,39]. MiSeq sequencing generated a total of 16,167,580 paired raw reads that, after quality control and chimera removal, resulted in a total of 12,722,738 processed paired-end V3-V4 reads, with an average read count of 76,643 reads per sample (min = 34,785; max = 133,199). After the removal of singletons, doubletons, rare Amplicon sequence variants (ASV; occurring in less than 5% of samples), and unassigned taxa, sequences could be assigned to 15,751 OTU (operational taxonomic units) in 83 samples. Of the identified 15,751 OTUs, 521 could be resolved at least to the genus level.

### Diversity and composition of the gut microbiome

**Fig 1:**
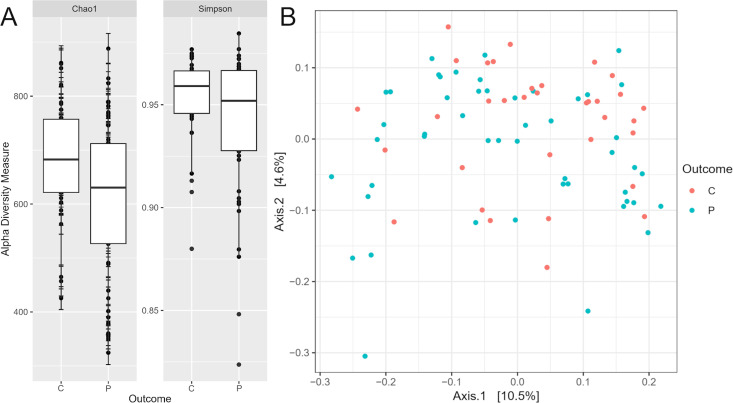
Alpha and beta diversity comparison of PD patients vs. healthy controls. (A) Alpha diversity of PD patients and controls. (B) Principal coordinate analysis (PCoA) plot generated by the Weighted Unifrac distance between PD patient (P) and healthy control (C) groups.

Our results showed a typically high gut microbial diversity in PD patients and controls alike. Microbial richness, as measured by alpha and beta diversity, showed no statistically significant difference, despite the median alpha diversity appearing slightly lower in the PD group than in the controls (Fig 1).

A similar relative abundance of bacterial phyla was observed in the gut microbiome of PD patients and healthy controls, with the dominant phyla being *Bacteroidota* (57.0%), *Bacillota* (formerly *Firmicutes*, 32.0%), and *Pseudomonadota* (5.0%), followed by *Actinomycetota, Fusobacteriota, Lentisphaerota, Synergistota, Verrucomicrobiota, Thermodesulfobacteriota*, at less than 1.0% of all bacteria present.

**Fig 2:**
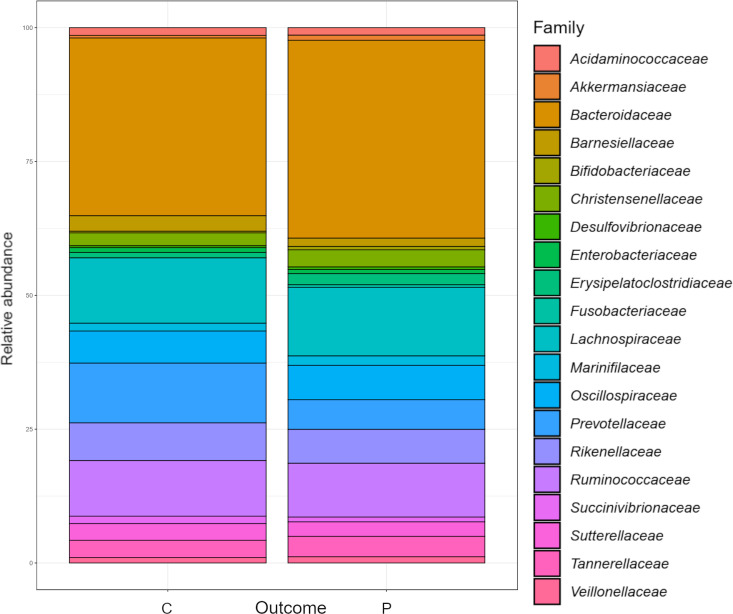
Relative abundance of phyla in the gut microbiome od PD patients and healthy controls. Relative abundance comparison of top 20 bacterial families in the gut microbiome of the PD patients (P) and healthy controls (C).

The family *Bacteroidaceae* represented 35.0% of total gut bacteria, followed by *Lachnospiraceae* (12.2%), *Ruminococcaceae* (10.0%), *Prevotellaceae* (7.4%), *Rikenellaceae* (6.6%), *Oscillospiraceae* (6.0%), *Tannerellaceae* (3.5%), *Sutterellaceae* (2.9%), *Christensenellaceae* (2.7%), *Barnesiellaceae* (2.2%), *Marinifilaceae* (1.6%), *Erysipelatoclostridiaceae* (1.4%), *Acidaminococcaceae* (1.3%),*Succinivibrionaceae* (1.2%), *Veillonellaceae* (0.9%), and remaining 60 families adding to the total of 5.1% of bacteria present. The relative abundance comparison of the top 20 microbial families in PD vs. healthy controls is shown in Fig 2.

### Differential relative abundance between PD patients and healthy controls

**Fig 3:**
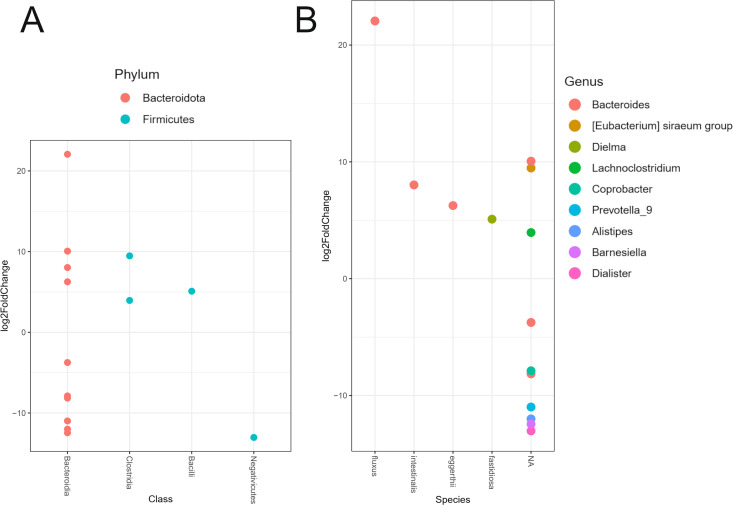
Differential relative abundance of gut bacteria in PD patients and healthy controls. (A) Differential relative abundance of phyla, and (B) genera and species in log2fold change between the PD patients and healthy controls.

The differential relative abundance analysis identified statistically significant differences in 9 genera from the phyla *Bacteroidota* and *Bacillota* (formerly *Firmicutes*), which was expected as these are the most prevalent and diverse phyla (Fig 3). In PD patients, *Bacteroides fluxus*, *B. intestinalis* and *B. eggerthii* were increased more than fivefold compared to cohorts, as was *Dielma fastidiosa*, which was only present in three of the controls and in 18 of the PD patients (in low numbers in all of these samples). On the other hand, genera *Alistipes*, *Barnesiella*, *Dialister* and *Prevotella_9* of unknown species, had more than 10-fold lower relative abundance in PD patients than controls (Fig 3).

## Discussion

We investigated the characteristics of the gut microbiome in drug-naïve PD patients and healthy controls by sequencing the V3-V4 region of the 16S rRNA.

### Microbiome diversity

While a recent meta-analysis has shown that alpha diversity may be increased in PD patients as opposed to healthy controls, which has been attributed to a decrease in the abundance of dominant bacterial species and an increase in the abundance of rare bacterial taxa [38], we found that microbial richness measured using alpha and beta diversity showed no statistically significant difference. The possible explanation for this is that contrary to previous studies, our study population is comprised of early phase drug-naïve patients and that the differences between patients and controls become more pronounced as the disease progresses and dopaminergic therapy is implemented.

### Relative abundance of individual taxa

In our study, the dominant taxa that showed a difference in relative abundance belonged to the phyla *Bacteroidota* and *Bacillota* (formerly *Firmicutes*), which was expected given that changed *Bacteroidota*/*Firmicutes* ratios have previously been observed in other studies [39]. The *Bacteroides fluxus*, *B. intestinalis* and *B. eggerthii* showed more than a fivefold increase in relative abundance in PD patients, while several unidentified additional *Bacteroides spp.* showed a decrease in abundance (Fig 3). Similarly, two studies previously identified *Bacteroides* of unknown species as having a decreased relative abundance in PD patients [40,41].

Previous studies have shown that relative abundance of *B. eggerthii* is increased in PD patients and was linked with hyposmia [42,43]. To our knowledge, neither *B. intestinalis* nor *B. fluxus* have individually been shown to be increased in PD. However, *B. intestinalis* has previously been correlated with neuro-inflammation, most notably in Alzheimer’s disease [44]. On the other hand, the abundance of *B. fluxus* was observed as increased in patients with sarcopenia [45], which has been shown to be more common in PD patients in certain instances than in the healthy population [46]. Other members of the *Bacteroides* genus, such as *B. fragilis*, have been similarly shown to have a higher relative abundance in PD patients [47].

Interestingly, while both the family *Rikenellaceae* [48] and the genus *Alistipes* were shown as increased in abundance in PD patients in previous studies [49,50] in our study *Alistipes spp.* showed a decrease in relative abundance. Whether this observed difference is due to the differences in study design, diet or therapy of the patients remains to be determined. Another member of the *Bacteroidota* phylum, which has continuously been shown to have a reduced relative abundance in PD as opposed to healthy controls is the family *Prevotellaceae*, in particular its genus *Prevotella* [51,52], with Aho et al. hypothesizing a link between this decrease with a faster-progressing disease phenotype [53]. In our study, a single member of this family, the ASV of genus *Prevotella_9* of unknown species, was shown to be decreased in drug-naïve PD patients, with no changes shown on the family level.

The second phylum in our study that showed a difference between PD patients and healthy controls was the *Bacillota* phylum (previously called *Firmicutes*), with its relative abundance being increased in PD compared to controls, which is in line with previous research. A member of the *Erysipelotrichaceae* family, the species *Dielma fastidiosa*, was found in several patients and only a few controls in our cohort. To our knowledge, the *Dielma* genus, named after a village in Dielmo, Senegal, has so far been described in a limited number of cases, both in a healthy individual and in a bacteraemia patient [54]. A study into IgA-biome profiles and their correlation with PD phenotype (akinetic or tremor dominant), has also identified *D. fastidiosa* in PD patients, but the species was not associated with a pro-inflammatory profile that could drive dysbiosis [39,55].

An unknown species of the genus *Dialister* (phylum *Bacillota*, family *Veillonellaceae*) was also identified as having a lower relative abundance. In line with our study, *Dialister invisus* has been shown to be lower in PD patients in the US population [55]. However, the genus *Dialister* has previously been shown to have a higher relative abundance in PD patients in a Southern China population [50], which may reflect dietary or other geographical differences.

The comparison of our findings with other available studies of the gut microbiome in drug-naïve PD patients, is limited by different study designs and aims. In a study conducted by Boertien et al., decreased levels of genera belonging to the family *Lachnospiraceae*, most notably *Roseburia*, have been reported, with a decreased relative abundance of *Christensenellaceae* and *Eggerthellaceae* in one of the two cohorts investigated [56], something which was previously also reported by Barichella et al. [57] but not replicated in our study. Another study, conducted by Melis et al., evaluated differences of the gut microbiome between drug-naïve PD patients and PD patients on levodopa (LD) and levodopa-carbidopa intestinal gel (LCIG), where it has been shown that there was an increase in the abundance of the taxa *Blautia* and *Lachnospirae* in LD patients as opposed to drug-naïve patients [58]. While this could point to the effects of levodopa on the gut microbiome, no healthy control was included in the study to better correlate potential differences between drug-naïve groups and the healthy population. With limited studies of this type this further points to a need for collection of drug-naïve microbiome datasets and correlations with healthy populations like in our study

The observed differences in our PD cohort could reflect multiple, early changes in gut composition which have previously been associated with PD. One of those is PD-related autonomic dysfunction which has been shown to lead to changes in gut motility and constipation, resulting in altered microbial niches which could in turn favor the growth of certain bacteria [59,60]. Another mechanism could be the α-synuclein aggregation in the enteric nervous system and gut mucosa, which could potentiate local inflammation, influencing microbial composition [61,62]. Changes in bile acid metabolism, along with mucus secretion, have also been implicated in PD, and could additionally shape the gut microbiome [51,63]. Additionally, SCFA have been implicated in gut dysbiosis. For instance, it has been shown that the reduction of SCFA-producing genera like *Alistipes* and *Barnesiella* may contribute to impaired gut barrier function and increased pro-inflammatory signaling [6,64]. Another study conducted by Kwon et. al, showed that specific dietary habits influence levels of SCFA bacteria, with a healthier diet increasing the levels of SCFA- producing bacteria, such as *Butyricicoccus*, *Coprococcus* *1*, and *Romboutsia*, while on the other hand, it has been shown that higher sugar intake lowered the levels of said taxa. The primary SCFA these bacteria produce is butyrate, which is beneficial for enteric function and its immunological integrity [65].

Interestingly, we did not observe a difference between these groups in our cohort, so it appears not to be the case at least in drug-naïve PD patients, such as ours, therefore additional, more focused studies are required to explore this. Another possible explanation for the lack of difference in the SCFA-producing bacteria could be our strict exclusion criteria, with which we attempted to minimize, or at least account for, the factors that are known to affect the microbiome. Indeed, our cohorts did not show significant differences in their diet, which was mostly the Mediterranean diet, previously shown to be beneficial in PD [66]. It is worth noting however, that in a systematic review by Kimble et al. no clear evidence of an effect of the Mediterranean diet on the gut microbiome was shown [67]. This could be due to methodological inconsistencies of the studies reviewed or the positive effect could be driven by a different mechanism. In our case, both PD patients and controls were matched in terms of diet, making the diet unlikely to introduce additional effects.

Additionally, we ensured both our patient group and control groups didn’t have pre-existing conditions that could affect intestinal motility and there was no antibiotic or probiotic usage involved.

Therefore, apart from the sample size, which was comparable to other studies currently conducted in this field, our chief limitation regarding the cohort was the observed age differences. So far, advanced age (60+) has been identified as a factor that could contribute to changes in microbiota composition with an increase of *Enterobacteriaceae* and *Bacteroidetes* and a decrease in *Bacillota* (formerly *Firmicutes*), *Bifidobacteria*, *Clostridium cluster XIV* and *Faecalibacterium* detected in older populations [68]. Other bacterial taxa have also been shown to change in composition such as *Clostridium* *bolteae*, *Escherichia* *coli*, *Escherichia* *unclassified*, *Parabacteroides* *distasonis*, *Parabacteroides unclassified*, and *Ruminococcus gnavus* [69]. However, in a study conducted by Bartosh et al. it has been found that in healthy older populations with no pronounced comorbidities, there were no significant changes in the gut microbiome when compared to younger populations, while in the elderly group that was hospitalized or was taking antibiotics, these changes were more apparent [70]. In the previously mentioned studies conducted by Petrov et al. and Vascellari et al., the patients were either age-matched [40] or there was a difference in mean age of almost twenty years [41], yet they showed similar results. Age-related changes in the gut microbiome could thus be interpreted as a result of individual factors, such as general physiological deterioration that leads to decreased mobility and thus also a decreased intestinal motility, while factors like diet and medication for various comorbidities, which could follow older age, could in turn further alter microbiome composition [71,72]. In conclusion, it is unlikely that the differences we have observed are due to the difference in age between the cohorts.

Our study potentially faces additional limitations regarding the interpretation of factors such as coffee consumption, alcohol consumption and smoking, due to various potential effects of these habits on the microbiome. An extensive investigation of the amount of coffee and alcohol consumption or smoking frequency was outside the scope of this study, however these variables were considered as they have previously been shown to possibly affect the microbiome.

Coffee consumption, for instance, has revealed a potentially positive effect on the gut microbiome, with moderate consumption (<4 cups a day) increasing the relative abundance of taxa such as *Baccillota* (formerly *Firmicutes*) and *Actinoctobacteria*, as well as *Bifidobacterium* *spp.* and *Enterobacteria*, with an effect on overall diversity in regular coffee drinkers, all which have been shown to be beneficial. High coffee consumption (>5 cups a day) has been linked with negative effects such as reflux disease [73].

Regarding alcohol consumption, research into its interplay with the microbiome of human subjects has been scarce, however it has been shown to have more of an effect on the gut microbiome in abuse of alcohol and alcohol use disorder [74] than in occasional and acute consumption, with the magnitude of effect being mostly minimal [75,76]. It is worth noting that the research into the way alcohol influences the human microbiome is scarce, making it harder to evaluate its overall effect on the microbiome.

It has been shown that smoking is associated with a decrease in beneficial bacteria such as *Bacillota* (formerly *Firmicutes*) and an increase in potentially harmful bacteria such as *Bacteroidetes* and *Proteobacteria*, as well as a decrease in overall diversity [77]. In our study, smoking was also significantly less reported in PD patients than in controls. How and whether this relates to PD currently remains unknown.

### Correlation of microbial abundance and clinical features of PD and potential for therapeutic intervention

As part of our initial screening, we evaluated our PD patients through UPDRS, NMSQ and MOCA (Table 1), since it has been previously found that the abundance of various microbial genera could correlate with PD symptom severity, albeit in a limited number of studies [20]. Positive correlation of relative abundance with motor symptom severity as expressed by UPDRS III scores was found with families *Lactobacillaceae* [49] and *Enterobacteriaceae* [78], genera such as *Flavonifractor, Peptococcus* [79], *Phascolarctobacterium, Coprococcus, Akkermansia* [49], *Clostridium cluster XIVa* [80], and species such as *Ruminoccoccus sp AM07–15* [81] and *E. coli* [82]. On the other hand, negative correlation between relative abundance and motor symptom severity was shown for families *Lachnospiraceae* [57]*, Prevotellaceae* [52,53], and genera *Blautia* [83]*, Prevotella* [53], and *Paraprevotella* [79]*.* Relative abundance was also positively correlated with non-motor symptom severity, such as in the case of cognitive decline as expressed by MMSE and MOCA scores. This was shown in the cases of the family *Lactobacillaceae* [49] and genera *Ruminococcus, Acidaminococcus, Barnesiella, Sutterella* and *Alistipes* [84]. A negative correlation between relative abundance and non-motor symptom severity has been found in the cases such as the family *Christensenellaceae* [57] and genera such as *Clostridium XIV b* and *Butyriococcus* [85].

How microbiota modulates PD symptoms and progression is still a matter of debate, and various mechanisms, such as the gut-brain axis retro-vagal transport [18], where it has been hypothesized that misfolded α-synuclein was transported from the intestines to the brain [86], or modulation by SCFA [81], have been proposed. Previously, a pro-inflammatory *Ruminococcuss spp.* was linked with higher plasma propionic levels, which showed a positive correlation with UPDRS III scores [81]. For example, the genus *Prevotella* on the other hand is associated with a higher butyric acid production, which has been hypothesized to postpone the age of onset in PD [53].

Changes in relative abundance of taxa identified in our study have previously been linked with various symptom severity in PD, such as in the case of phylum *Bacillota* (formerly *Firmicutes*), members of classes *Clostridia, Bacilli and Negativicutes* [79,81,84,87,88], as well as the genera *Bacteroides* [89], *Barnesiella* and *Alistipes*, [58]. Further analysis down the line should be conducted in our dataset to see whether or not similar changes would be found in our cohort, especially after including PD therapy.

Dopaminergic medication has previously been linked with changes in the microbiome, with certain taxa, such as *E. faecalis*, being more pronounced in moderate responders to levodopa than in good responders [90]. In other cases, it has been shown that *H. pylori* not only has an effect on symptom severity but also on levodopa availability [91]. A major strength of our study is the inclusion of only drug-naïve patients, which removes therapy as a possible major confounder considering the oral administration of medication in all first-line therapies for PD. By clearly identifying therapy-associated taxa in PD patients, it may be possible to anticipate the therapeutic response depending on the gut microbiome “type” in the future, as well as influence the microbiome through the use of probiotics, prebiotics or microbial transplantation [11,92]. Multiple studies have been conducted to examine the possible benefits of probiotics in PD, with positive effects on motor and non-motor symptoms such as constipation with *Lactobacillus* or *Bifidobacterium* species mostly used in various combinations [93]. Fecal microbiome transplant (FMT) has also been researched as a potential intervention for PD through various routes, including capsules administered orally [94,95], nasojejunally [96] or via colonoscopy [97]. However, while initial studies showed positive results, recently, the benefit of FMT was called into question by a double-blind placebo study [97], and therefore the clinical benefit of FTM remains inconclusive at best. Antibiotics have also been researched for microbiome-related treatment in PD, with examples such as rifampicin and doxycycline, which demonstrated reduction of alpha-synuclein oligomers forming fibrils in vitro [98]. Novel technologies such as CRISPR-based interventions and bacteriophages are also being researched [99]. With advancing technologies and ongoing research, the gut–brain axis holds promise as a key target for adjunctive therapy and supportive care in PD and extensive research in this field could improve overall care of people with PD.

## Conclusions

To conclude, our study shows that drug-naïve PD patients do not yet show significant differences in alpha diversity compared to healthy controls, something which has been previously observed in patients with therapy, while the relative abundance comparisons showed several differences observed in previous research. We observed differences in relative abundance of members of the phyla *Bacteroidetes* and *Bacillota* (formerly *Firmicutes*) between PD patients and healthy controls. Species *Bacteroides fluxus*, *B*. *intestinalis*, *B*. *eggerhii* and *Dielma fastidiosa* demonstrated an increase in relative abundance in PD patients as opposed to healthy controls, whereas we observed a decrease in relative abundance of *Alistipes*, *Barnesiella,* and *Dialister*, of unknown species. Our work is the first of its kind in the Croatian population and represents a foundational step for future longitudinal follow-up of the stool microbiome during PD progression.

## Supporting information

S1 Table Questionnaire on lifestyle habits (in Croatian). (DOCX)
