# Supplementary material for: Microbial diversity in drug-naïve Parkinson’s disease patients
Source: PLoS One. 2025 Aug 18;20(8):e0328761. doi: 10.1371/journal.pone.0328761 (PMC12360607; doi:10.1371/journal.pone.0328761)
Supplement: S1 Table — (DOCX) [file pone.0328761.s001.docx]

| OSNOVNI PODATCI | | | | | | | | | | | | | | | **Šifra:** | | | | | | | | | | | | | | | | | | | | | |  | | | | | | | |
| --- | --- | --- | --- | --- | --- | --- | --- | --- | --- | --- | --- | --- | --- | --- | --- | --- | --- | --- | --- | --- | --- | --- | --- | --- | --- | --- | --- | --- | --- | --- | --- | --- | --- | --- | --- | --- | --- | --- | --- | --- | --- | --- | --- | --- |
| **Prezime** | | | | | | **Spol** | | | **Datum rođenja** | | | | | | | | | | | | | **Mjesto rođenja** | | | | | | | | | | | | | | | | **Dob** | | | | | | |
|  | | | | | |  | | |  | | | | | | | | | | | | |  | | | | | | | | | | | | | | | |  | | | | | | |
| **PREHRAMBENE NAVIKE** | | | | | | | | | | | | | | | | | | | | | | | | | | | | | | | | | | | | | | | | | | | | |
| Kako sami ocjenjujete svoje prehrambene navike? | | | | | | | | | | | | | | | | | | | | | | | | | | | | | | | | | | | | | | | | | | | | |
| Jedem sve vrste hrane | | | | | | | | | | Da ne | | | | | | | | | | | Jedem vegetarijanski | | | | | | | | | | | | | | | | | | | | | | | Da ne |
| Jedem sve osim crvenog mesa | | | | | | | | | | Da ne | | | | | | | | | | | Jaja | | | | | | | | | | | | | | | | | | | | | | | Da ne |
| Jedem sve osim ribe | | | | | | | | | | Da ne | | | | | | | | | | | Mlijeko/mliječni proizvodi | | | | | | | | | | | | | | | | | | | | | | | Da ne |
| Jedem veganski | | | | | | | | | | Da ne | | | | | | | | | | | Ribe | | | | | | | | | | | | | | | | | | | | | | | Da ne |
| Jedem nemasno | | | | | | | | | | Da ne | | | | | | | | | | | Drugo: | | | | | | |  | | | | | | | | | | | | | | | | |
| **Koliko često ste protekloga tjedna jeli/pili?** | | | | | | | | | | | | | | | | | | | | | | | | | | | | | | | | | | | | | | | | | | | | |
|  | | | | | | | | Nijednom | | | | | | | | 1-2x tjedno | | | | | | | | | | | 3-5 x tjedno | | | | | | | | | | | | | | 6-7 x tjedno | | | |
| Krumpir | | | | | | | |  | | | | | | | |  | | | | | | | | | | |  | | | | | | | | | | | | | |  | | | |
| Riža, tjestenina | | | | | | | |  | | | | | | | |  | | | | | | | | | | |  | | | | | | | | | | | | | |  | | | |
| Pahuljice, žitarice, muesli | | | | | | | |  | | | | | | | |  | | | | | | | | | | |  | | | | | | | | | | | | | |  | | | |
| Mlijeko i mliječni proizvodi | | | | | | | |  | | | | | | | |  | | | | | | | | | | |  | | | | | | | | | | | | | |  | | | |
| Ribe | | | | | | | |  | | | | | | | |  | | | | | | | | | | |  | | | | | | | | | | | | | |  | | | |
| Bijelo meso | | | | | | | |  | | | | | | | |  | | | | | | | | | | |  | | | | | | | | | | | | | |  | | | |
| Crvene meso | | | | | | | |  | | | | | | | |  | | | | | | | | | | |  | | | | | | | | | | | | | |  | | | |
| Svježe povrće | | | | | | | |  | | | | | | | |  | | | | | | | | | | |  | | | | | | | | | | | | | |  | | | |
| Kuhano povrće | | | | | | | |  | | | | | | | |  | | | | | | | | | | |  | | | | | | | | | | | | | |  | | | |
| Svježe voće | | | | | | | |  | | | | | | | |  | | | | | | | | | | |  | | | | | | | | | | | | | |  | | | |
| Kuhano voće, kompot, marmelada | | | | | | | |  | | | | | | | |  | | | | | | | | | | |  | | | | | | | | | | | | | |  | | | |
| Slatkiši | | | | | | | |  | | | | | | | |  | | | | | | | | | | |  | | | | | | | | | | | | | |  | | | |
| Gazirana pića | | | | | | | |  | | | | | | | |  | | | | | | | | | | |  | | | | | | | | | | | | | |  | | | |
| Jaja | | | | | | | |  | | | | | | | |  | | | | | | | | | | |  | | | | | | | | | | | | | |  | | | |
| Kava | | | | | | | |  | | | | | | | |  | | | | | | | | | | |  | | | | | | | | | | | | | |  | | | |
| Crni čaj | | | | | | | |  | | | | | | | |  | | | | | | | | | | |  | | | | | | | | | | | | | |  | | | |
| Voćni čaj | | | | | | | |  | | | | | | | |  | | | | | | | | | | |  | | | | | | | | | | | | | |  | | | |
| Alkoholna pića | | | | | | | |  | | | | | | | |  | | | | | | | | | | |  | | | | | | | | | | | | | |  | | | |
| Bijeli šećer | | | | | | | |  | | | | | | | |  | | | | | | | | | | |  | | | | | | | | | | | | | |  | | | |
| **Ako pijete kavu ili crni čaj, koliko šalica dnevno popijete?** | | | | | | | | | | | | | | | | | | | | | | | | | | | | | | | | | | | | | | | | | | | | |
| **Kava:** | | |  | | | | | | | | | | | | | | | **Crni čaj:** | | | | | | | | | | | | | | |  | | | | | | | | | | | |
| **Koliko šnita kruha obično pojedete dnevno?** | | | | | | | | | | | | | | | | | | | | | | | | | | | | | | | | | | | | | | | | | | | | |
| **Bijeli kruh:** | | |  | | | | | | | | | | | | | | | **Crni kruh:** | | | | | | | | | | | | | | |  | | | | | | | | | | | |
| **Solite li već pripremljenu hranu?** | | | | | | | | | | | | | | | | | | | | | | | | | | | | | | | | | | | | | | | | | | | | |
| **uvijek** | | **Često** | | | | | | | | | **ponekad** | | | | | | | | | | | | | **rijetko** | | | | | | | | | | | | | | | **Nikad** | | | | | |
| **Upotrebljavate li umjetna sladila?** | | | | | | | | | | | | | | | | | | | | | | | | | | | | | | | | | | | | | | | | | | | | |
| **uvijek** | | **Često** | | | | | | | | | **ponekad** | | | | | | | | | | | | | **rijetko** | | | | | | | | | | | | | | | **Nikad** | | | | | |
| **Uzimate li nadomjeske prehrani, vitamine, minerale ili probiotike sa ili bez recepta?** | | | | | | | | | | | | | | | | | | | | | | | | | | | | | | | | | | | | | | | **Da ne** | | | | | |
| **Ime nadomjeska prehrani** | | | | | | | | | | | | | | | | | | | | **Učestalost uzimanja** | | | | | | | | | | | | | | | | | | | | | | | | |
|  |  |  |  |  |  |  |  |  |  |  |  |  |  |  |  |  |  |  |  | **dnevno** | | | | | | | | | | **tjedno** | | | | | | | | | | | | **Mjesečno** | | |
| **1.** |  | | | | | | | | | | | | | | | | | | |  | | | | | | | | | |  | | | | | | | | | | | |  | | |
| **2.** |  | | | | | | | | | | | | | | | | | | |  | | | | | | | | | |  | | | | | | | | | | | |  | | |
| **3.** |  | | | | | | | | | | | | | | | | | | |  | | | | | | | | | |  | | | | | | | | | | | |  | | |
| **4.** |  | | | | | | | | | | | | | | | | | | |  | | | | | | | | | |  | | | | | | | | | | | |  | | |
| **5.** |  | | | | | | | | | | | | | | | | | | |  | | | | | | | | | |  | | | | | | | | | | | |  | | |
| **6.** |  | | | | | | | | | | | | | | | | | | |  | | | | | | | | | |  | | | | | | | | | | | |  | | |
| **7.** |  | | | | | | | | | | | | | | | | | | |  | | | | | | | | | |  | | | | | | | | | | | |  | | |
| **8.** |  | | | | | | | | | | | | | | | | | | |  | | | | | | | | | |  | | | | | | | | | | | |  | | |
| **9.** |  | | | | | | | | | | | | | | | | | | |  | | | | | | | | | |  | | | | | | | | | | | |  | | |
| **10.** |  | | | | | | | | | | | | | | | | | | |  | | | | | | | | | |  | | | | | | | | | | | |  | | |
| **Druge napomene:** | | | | |  | | | | | | | | | | | | | | | | | | | | | | | | | | | | | | | | | | | | | | | |
| **PUŠENJE** | | | | | | | | | | | | | | | | | | | | | | | | | | | | | | | | | | | | | | | | | | | | |
| **Jeste li ikada pušili u životu?** | | | | | | | | | | | | | | | | | | | | | | | | | | | | | | | | | | **Da Ne** | | | | | | | | | | |
| **Upotrebljavate li redovito duhanske proizvode?** | | | | | | | | | | | | | | | | | | | | | | | | | | | | | | | | | | **Da Ne** | | | | | | | | | | |
| **S koliko ste godina počeli pušiti?** | | | | | | | | | | | | | | | | | | | | | | | | | | | | | | | | | |  | | | | | | | | | | |
| **Pušite li još?** | | | | | | | | | | | | | | | | | | | | | | | | | | | | | | | | | | **Da Ne** | | | | | | | | | | |
| **Ako ste prestali pušiti, s koliko godina ste prestali pušiti?** | | | | | | | | | | | | | | | | | | | | | | | | | | | | | | | | | |  | | | | | | | | | | |
| **Koliko godina ste pušili/pušite?** | | | | | | | | | | | | | | | | | | | | | | | | | | | | | | | | | |  | | | | | | | | | | |
| **Koje duhanske proizvode trenutno koristite?** | | | | | | | | | | | | | | | | | | **dnevno** | | | | | | | | | | | **tjedno** | | | | | | | | | | | | | **mjesečno** | | |
| **Cigarete s filtrom** | | | | | | | | **Da ne** | | | | | | | | | | |  | | | | | | | | | |  | | | | | | | | | | | | |  | | |
| **Cigare bez filtera** | | | | | | | | **Da ne** | | | | | | | | | | |  | | | | | | | | | |  | | | | | | | | | | | | |  | | |
| **E-cigarete** | | | | | | | | **Da ne** | | | | | | | | | | |  | | | | | | | | | |  | | | | | | | | | | | | |  | | |
| **Lula** | | | | | | | | **Da ne** | | | | | | | | | | |  | | | | | | | | | |  | | | | | | | | | | | | |  | | |
| **Cigara** | | | | | | | | **Da ne** | | | | | | | | | | |  | | | | | | | | | |  | | | | | | | | | | | | |  | | |
| **Drugo:** | | | | | | | |  | | | | | | | | | | |  | | | | | | | | | |  | | | | | | | | | | | | |  | | |
| **Koje duhanske proizvode ste koristili dok ste pušili?** | | | | | | | | | | | | | | | | | | **dnevno** | | | | | | | | | | | **tjedno** | | | | | | | | | | | | | **mjesečno** | | |
| **Cigarete s filtrom** | | | | | | | | **Da ne** | | | | | | | | | | |  | | | | | | | | | |  | | | | | | | | | | | | |  | | |
| **Cigare bez filtera** | | | | | | | | **Da ne** | | | | | | | | | | |  | | | | | | | | | |  | | | | | | | | | | | | |  | | |
| **E-cigarete** | | | | | | | | **Da ne** | | | | | | | | | | |  | | | | | | | | | |  | | | | | | | | | | | | |  | | |
| **Lula** | | | | | | | | **Da ne** | | | | | | | | | | |  | | | | | | | | | |  | | | | | | | | | | | | |  | | |
| **Cigara** | | | | | | | | **Da ne** | | | | | | | | | | |  | | | | | | | | | |  | | | | | | | | | | | | |  | | |
| **Drugo:** | | | | | | | |  | | | | | | | | | | |  | | | | | | | | | |  | | | | | | | | | | | | |  | | |
| **PASIVNO PUŠENJE** | | | | | | | | | | | | | | | | | | | | | | | | | | | | | | | | | | | | | | | | | | | | |
| **Jeste ikada bili izloženi pasivnom pušenju?** | | | | | | | | | | | | | | | | | | | | | | | | | | | | | | | | | | **Da Ne** | | | | | | | | | | |
| **Jeste li trenutno izloženi pasivnom pušenju?** | | | | | | | | | | | | | | | | | | | | | | | | | | | | | | | | | | **Da Ne** | | | | | | | | | | |
| **Koliko ste godina, ako ste bili, izloženi pasivnom pušenju?** | | | | | | | | | | | | | | | | | | | | | | | | | | | | | | | | | |  | | | | | | | | | | |
| **Gdje ste bili izloženi pasivnom pušenju?** | | | | | | | | | | | | | | | | | | | | | | | | | | | | | | | | | | | | | | | | | | | | |
| **kući** | | | | **Na poslu** | | | | | | | | | **U slobodno vrijeme** | | | | | | | | | | | | | | | | | | | | | **Drugo** | | | | | | | | | | |
| **Po vašoj procjeni, koliko ste bili izloženi?** | | | | | | | | | | | | **sati dnevno** | | | | | | | | | | | | | **Dana tjedno** | | | | | | | | | | | | | | | **Dani mjesečno** | | | | |
|  |  |  |  |  |  |  |  |  |  |  |  |  | | | | | | | | | | | | |  | | | | | | | | | | | | | | |  | | | | |
| **Druge napomene:** | | | | | | | | | | | |  | | | | | | | | | | | | | | | | | | | | | | | | | | | | | | | | |
| **UPORABA ALKOHOLA** | | | | | | | | | | | | | | | | | | | | | | | | | | | | | | | | | | | | | | | | | | | | |
| **Jeste li ikada u životu probali alkohol?** | | | | | | | | | | | | | | | | | | | | | | | | | | | | | | | | | | **Da Ne** | | | | | | | | | | |
| **Ako jeste, u kojoj dobi ste počeli upotrebljavati alkohol?** | | | | | | | | | | | | | | | | | | | | | | | | | | | | | | | | | |  | | | | | | | | | | |
| **Upotrebljavate li još uvijek alkoholna pića?** | | | | | | | | | | | | | | | | | | | | | | | | | | | | | | | | | | **Da Ne** | | | | | | | | | | |
| **Upotrebljavate li redovito alkoholna pića?** | | | | | | | | | | | | | | | | | | | | | | | | | | | | | | | | | | **Da Ne** | | | | | | | | | | |
| **Ako ste prestali, s koliko godina ste prestali?** | | | | | | | | | | | | | | | | | | | | | | | | | | | | | | | | | |  | | | | | | | | | | |
| **Koliko godina ste ukupno upotrebljavali alkoholna pića?** | | | | | | | | | | | | | | | | | | | | | | | | | | | | | | | | | |  | | | | | | | | | | |
| **Koja alkoholna pića trenutno konzumirate?** | | | | | | | | | | | | | | | | | | | | | | | | | | | | | | | | | | | | | | | | | | | | |
| **Piće** | | | | | | | | **Količina** | | | | | | **Upotreba** | | | | | | | | | **Dnevno** | | | | | | | | **Tjedno** | | | | | | | | | | | | **Mjesečno** | |
| **Pivo** | | | | | | | | **500 ml** | | | | | | **Da ne** | | | | | | | | |  | | | | | | | |  | | | | | | | | | | | |  | |
| **Vino** | | | | | | | | **100 ml** | | | | | | **Da ne** | | | | | | | | |  | | | | | | | |  | | | | | | | | | | | |  | |
| **Desertno vino, 20% vol** | | | | | | | | **40 ml** | | | | | | **Da ne** | | | | | | | | |  | | | | | | | |  | | | | | | | | | | | |  | |
| **Viski, konjak, 40% vol** | | | | | | | | **40 ml** | | | | | | **Da ne** | | | | | | | | |  | | | | | | | |  | | | | | | | | | | | |  | |
| **Drugo:** | | | | | | | |  | | | | | |  | | | | | | | | |  | | | | | | | |  | | | | | | | | | | | |  | |
| **Koja alkoholna pića ste ranije konzumirali?** | | | | | | | | | | | | | | | | | | | | | | | | | | | | | | | | | | | | | | | | | | | | |
| **Piće** | | | | | | | **količina** | | | | | **Upotreba** | | | | | | | | | | | **Dnevno** | | | | | | | | **Tjedno** | | | | | | | | | | | | **Mjesečno** | |
| **Pivo** | | | | | | | | **500 ml** | | | | | | **Da ne** | | | | | | | | |  | | | | | | | |  | | | | | | | | | | | |  | |
| **Vino** | | | | | | | | **100 ml** | | | | | | **Da ne** | | | | | | | | |  | | | | | | | |  | | | | | | | | | | | |  | |
| **Desertno vino, 20% vol** | | | | | | | | **40 ml** | | | | | | **Da ne** | | | | | | | | |  | | | | | | | |  | | | | | | | | | | | |  | |
| **Viski, konjak, 40% vol** | | | | | | | | **40 ml** | | | | | | **Da ne** | | | | | | | | |  | | | | | | | |  | | | | | | | | | | | |  | |
| **Drugo:** | | | | | | | |  | | | | | |  | | | | | | | | |  | | | | | | | |  | | | | | | | | | | | |  | |
| **DRUGE PSIHOAKTIVNE TVARI (na recept ili bez)** | | | | | | | | | | | | | | | | | | | | | | | | | | | | | | | | | | | | | | | | | | | | |
| **Jeste li ikad u životu uzeli neku psihoaktivnu tvar, izuzev alkohola i duhana?** | | | | | | | | | | | | | | | | | | | | | | | | | | | | | | | | | | | **Da ne** | | | | | | | | | |
| **Ako jeste, u kojoj ste ih dobi uzeli prvi puta?** | | | | | | | | | | | | | | | | | | | | | | | | | | | | | | | | | | |  | | | | | | | | | |
| **Upotrebljavate li još uvijek te tvari?** | | | | | | | | | | | | | | | | | | | | | | | | | | | | | | | | | | | **Da ne** | | | | | | | | | |
| **Ako ste prestali, u kojoj dobi ste prestali?** | | | | | | | | | | | | | | | | | | | | | | | | | | | | | | | | | | | |  | | | | | | | | |
| **Koliko ste ih ukupno godina koristili?** | | | | | | | | | | | | | | | | | | | | | | | | | | | | | | | | | | | |  | | | | | | | | |
| **Molimo označite ako ste navedene supstance uzimali za ublažavanje simptoma vaše bolesti ili rekreativno?** | | | | | | | | | | | | | | | | | | | | | | | | | | | | | | | | | | | | | | | | | | | | |
| **Ime psihoaktivne tvari** | | | | | | | | | | **Samo 1x** | | | | | | | **Povremeno godišnje** | | | | | | | | | **Redovito,** | | | | | | | | | | | | | | | | | | |
|  |  |  |  |  |  |  |  |  |  |  |  |  |  |  |  |  |  |  |  |  |  |  |  |  |  | **dnevno** | | | | | | **tjedno** | | | | | | | | | | | | **Mjesečno** |
| **Amfetamini** | | | | | | | | | |  | | | | | | |  | | | | | | | | |  | | | | | |  | | | | | | | | | | | |  |
| **Barbituarati** | | | | | | | | | |  | | | | | | |  | | | | | | | | |  | | | | | |  | | | | | | | | | | | |  |
| **Benzodiazepini (Apaurin, Lexaurin, Dormicum** | | | | | | | | | |  | | | | | | |  | | | | | | | | |  | | | | | |  | | | | | | | | | | | |  |
| **DXM** | | | | | | | | | |  | | | | | | |  | | | | | | | | |  | | | | | |  | | | | | | | | | | | |  |
| **Ekstazi** | | | | | | | | | |  | | | | | | |  | | | | | | | | |  | | | | | |  | | | | | | | | | | | |  |
| **Efedrin ili pseudoefedrin** | | | | | | | | | |  | | | | | | |  | | | | | | | | |  | | | | | |  | | | | | | | | | | | |  |
| **GHB** | | | | | | | | | |  | | | | | | |  | | | | | | | | |  | | | | | |  | | | | | | | | | | | |  |
| **Heroin** | | | | | | | | | |  | | | | | | |  | | | | | | | | |  | | | | | |  | | | | | | | | | | | |  |
| **Ketamin** | | | | | | | | | |  | | | | | | |  | | | | | | | | |  | | | | | |  | | | | | | | | | | | |  |
| **Kokain** | | | | | | | | | |  | | | | | | |  | | | | | | | | |  | | | | | |  | | | | | | | | | | | |  |
| **Marihuana / Konoplja** | | | | | | | | | |  | | | | | | |  | | | | | | | | |  | | | | | |  | | | | | | | | | | | |  |
| **Meskalin** | | | | | | | | | |  | | | | | | |  | | | | | | | | |  | | | | | |  | | | | | | | | | | | |  |
| **Mefedron** | | | | | | | | | |  | | | | | | |  | | | | | | | | |  | | | | | |  | | | | | | | | | | | |  |
| **Metadon** | | | | | | | | | |  | | | | | | |  | | | | | | | | |  | | | | | |  | | | | | | | | | | | |  |
| **Morfij** | | | | | | | | | |  | | | | | | |  | | | | | | | | |  | | | | | |  | | | | | | | | | | | |  |
| **Nexus** | | | | | | | | | |  | | | | | | |  | | | | | | | | |  | | | | | |  | | | | | | | | | | | |  |
| **Opij** | | | | | | | | | |  | | | | | | |  | | | | | | | | |  | | | | | |  | | | | | | | | | | | |  |
| **PCP** | | | | | | | | | |  | | | | | | |  | | | | | | | | |  | | | | | |  | | | | | | | | | | | |  |
| **Psilocibin** | | | | | | | | | |  | | | | | | |  | | | | | | | | |  | | | | | |  | | | | | | | | | | | |  |
| **Tramadol, npr. Tramal** | | | | | | | | | |  | | | | | | |  | | | | | | | | |  | | | | | |  | | | | | | | | | | | |  |
| **Napomene:** | | | | | | | | | |  | | | | | | | | | | | | | | | | | | | | | | | | | | | | | | | | | | |
| **UPOTREBA KONOPLJE / MARIHUANE I NJENIH EKSTRAKATA**  **(Npr. za ublažavanje simptoma Parkinsonove bolesti)** | | | | | | | | | | | | | | | | | | | | | | | | | | | | | | | | | | | | | | | | | | | | |
| **Jeste li ikada probali konoplju?** | | | | | | | | | | | | | | | | | | | | | | | | | | | | | | | | | | **Da Ne** | | | | | | | | | | |
| **Ako da, u kojoj dobi?** | | | | | | | | | | | | | | | | | | | | | | | | | | | | | | | | | |  | | | | | | | | | | |
| **Upotrebljavate li još uvijek konoplju?** | | | | | | | | | | | | | | | | | | | | | | | | | | | | | | | | | | **Da Ne** | | | | | | | | | | |
| **Ako da, upotrebljavate li konoplju redovito?** | | | | | | | | | | | | | | | | | | | | | | | | | | | | | | | | | | **Da Ne** | | | | | | | | | | |
| **Ako ste prestali, koliko ste imali godina kada ste prestali?** | | | | | | | | | | | | | | | | | | | | | | | | | | | | | | | | | |  | | | | | | | | | | |
| **Koliko ste godina ukupno upotrebljavali konoplju?** | | | | | | | | | | | | | | | | | | | | | | | | | | | | | | | | | |  | | | | | | | | | | |
| **U kojem obliku trenutno upotrebljavate konoplju?** | | | | | | | | | | | | | | | | | | | | | | | | | | | | | | | | | | | | | | | | | | | | |
| **Oblik** | | | | | | | | | | | | | | **Upotreba** | | | | | | | | | **Dnevno** | | | | | | | | **Tjedno** | | | | | | | | | | | | **Mjesečno** | |
| **Ekstrakt u ulju** | | | | | | | | | | | | | | **Da ne** | | | | | | | | |  | | | | | | | |  | | | | | | | | | | | |  | |
| **Smola** | | | | | | | | | | | | | | **Da ne** | | | | | | | | |  | | | | | | | |  | | | | | | | | | | | |  | |
| **Mast** | | | | | | | | | | | | | | **Da ne** | | | | | | | | |  | | | | | | | |  | | | | | | | | | | | |  | |
| **Cigareta / džoint** | | | | | | | | | | | | | | **Da ne** | | | | | | | | |  | | | | | | | |  | | | | | | | | | | | |  | |
| **Vaporizator** | | | | | | | | | | | | | | **Da ne** | | | | | | | | |  | | | | | | | |  | | | | | | | | | | | |  | |
| **Sa CBD** | | | | | | | | | | | | | | **Da ne** | | | | | | | | |  | | | | | | | |  | | | | | | | | | | | |  | |
| **Drugo:** | | | | | | | | | | | | | |  | | | | | | | | |  | | | | | | | |  | | | | | | | | | | | |  | |
| **U kojem obliku ste ranije upotrebljavali konoplju?** | | | | | | | | | | | | | | | | | | | | | | | | | | | | | | | | | | | | | | | | | | | | |
| **Oblik** | | | | | | | | | | | | | | **Upotreba** | | | | | | | | | **Dnevno** | | | | | | | | **Tjedno** | | | | | | | | | | | | **Mjesečno** | |
| **Ekstrakt u ulju** | | | | | | | | | | | | | | **Da ne** | | | | | | | | |  | | | | | | | |  | | | | | | | | | | | |  | |
| **Smola** | | | | | | | | | | | | | | **Da ne** | | | | | | | | |  | | | | | | | |  | | | | | | | | | | | |  | |
| **Mast** | | | | | | | | | | | | | | **Da ne** | | | | | | | | |  | | | | | | | |  | | | | | | | | | | | |  | |
| **Cigareta / džoint** | | | | | | | | | | | | | | **Da ne** | | | | | | | | |  | | | | | | | |  | | | | | | | | | | | |  | |
| **Vaporizator** | | | | | | | | | | | | | | **Da ne** | | | | | | | | |  | | | | | | | |  | | | | | | | | | | | |  | |
| **Sa CBD** | | | | | | | | | | | | | | **Da ne** | | | | | | | | |  | | | | | | | |  | | | | | | | | | | | |  | |
| **Drugo:** | | | | | | | | | | | | | |  | | | | | | | | |  | | | | | | | |  | | | | | | | | | | | |  | |
|  | | | | | | | | | | | | | | | | | | | | | | | | | | | | | | | | | | | | | | | | | | | | |
| **Dodatne napomene:** | | | | | | | | | | | | | |  | | | | | | | | | | | | | | | | | | | | | | | | | | | | | | |
